# Quantifying construction vibration effects on daily radiotherapy treatments

**DOI:** 10.1002/acm2.12386

**Published:** 2018-07-07

**Authors:** Jonathan J. Hindmarsh, Wendy L. Smith

**Affiliations:** ^1^ Tom Baker Cancer Centre Foothills Hospital 1331 29th St. N.W Calgary AB T2N 4N2

**Keywords:** construction, linear accelerators, vibration

## Abstract

The existing two‐story parkade is being replaced by a four‐story parkade on a hospital campus. The parkade is across a two‐lane access road from a cancer center with a nine‐linear accelerator radiotherapy department in the basement. The new parkade is supported by over 280 drilled and cased pilings installed at depths between 10 and 25 m depending on the underlying soil strata and varying diameters, up to 1.5 m. The construction work in such close proximity to the radiation therapy department resulted in significant vibrations being felt in the simulation and treatment vaults. The amplitude and frequency of the vibration was measured. Using vendor supplied documentation, the total vibratory amplitude of the linear accelerators in use within the department was calculated. The results fell outside of specification, resulting in changes to the way the project preceded following discussion with the project management team.

## 1 INTRODUCTION

Construction and development projects are not uncommon around hospitals and cancer centers. Construction affects in the hospital setting can be significant, from changes to air quality, patient and visitor access and flow, noise, and vibrations. Hospitals cannot simply shut down or move operations during constructions, and ongoing patient care must be prioritized over constructions issues. These effects make managing construction in a hospital environment particularly challenging.

In Fall 2016, work began to replace the existing two‐story parkade with a four‐story parkade. The above‐ground design of the new parkade requires over 280 drilled and cased pilings to be installed at depths between 10 and 25 m depending on the underlying soil strata and varying diameters, up to 1.5 m. The method chosen to install the piles was a vibration hammer: the basic procedure is to vibration hammer a steel sheath (casing) to the desired depth, auger the dirt out of the sheath, create a bell at the bottom, install reinforcing steel cages in the shaft, pour the concrete, and, finally, use the vibration unit to remove the sheath immediately after the concrete is poured. This allows the concrete to settle in the shaft and bind with the soil around it.

The vibrations from this construction method were transmitted through the ground to the basement‐level radiotherapy department at the adjacent cancer center. Vibration transmission was complex, but as the construction team starting working on casings closer to the center, the effects increased. During the fourth quarter of 2016, staff of the cancer center reported feeling periods of light vibrations in the treatment bunkers through their feet and patients reported feeling vibrations while lying on the treatment couch. The periods of vibrations varied in length from seconds to tens of minutes but were generally separated by hours or days of no vibratory activity. Over 3–4 weeks, the intensity of the vibrations gradually increased from being an annoyance to causing books to fall from shelves and causing pole mounted setup lasers to visibly shake at isocenter.

A second source of problematic vibrations was observed during the first quarter 2017 when a vibratory compactor was used across the road from the cancer center (<50 m from closest bunkers) to compact backfill. Following excavation, there is the requirement to fill and compact these areas; this was to be accomplished using vibratory compactors in one of three sizes with the smallest being a 500‐lb remote controlled unit to a full sized unit with an 8000‐lb compactor.

A search of the literature for the effect of vibrations on linear accelerators or computed tomography (CT) scanners turned up only one paper that tested a Siemens CT (Siemens, DE) and a Siemens mobile C‐arm cardiac angiography system^1^ in which it was found that there was no clinically significant degradation in image quality due to vibrations less than the specified particle velocity tolerance of 25 μm/s. There are numerous papers in the engineering literature that discuss the vibrations caused by piling,^2^ the potential for damage to nearby structures^3^, ^4^ and methods to predict and control ground vibrations.^5^ The limitation of the majority of these papers is that they deal with damage to residential structures for which the tolerance limit specified by Athanasopoulos^3^ is 5 mm/s (peak particle velocity). The limit for damage is 12.5 times higher than what Gordon^6^ gives as the comfort limit for generic office spaces, 400 μm/s (RMS velocity), and at least 50 times higher than what Ungar^7^ gives as the root mean square (RMS) particle velocity limit for vibrations in hospitals; 100 μm/s for general areas of the hospital and 25 μm/s for sensitive surgical areas such as those performing neurosurgery or microsurgery. Also included in Ungar's paper is a summary of the criteria provided by MRI equipment suppliers, which are generally equivalent to or stricter than the general hospital constraints.

Vibration limits depend significantly on the tolerance specified and the regulatory document. For the majority of articles, the tolerance criterion is damage (either structural or superficial). This contrasts to the International Standards Organisation (ISO) and American National Standards Institute (ANSI) criterion cited by Gordon^6^ and Ungar^7^ for which the tolerance criterion used is human exposure or equipment operational limits. Gordon^6^ provides a useful table containing the criterion category, maximum RMS particle velocity in μm/s and a description of where the category would apply. The categories are given descriptive names where they apply to human exposure limits, for more sensitive design constraints five vibration categories (VC) are used with designations of A to E specifying RMS particle velocities decreasing from 50 to 3 μm/s, respectively. For example, the residential day category specifies a maximum RMS particle velocity of 200 μm/s and is appropriate for sensitive sleep areas and most low powered microscopes, and the VC‐D category specifies a maximum RMS particle velocity of 6 μm/s and applies to electron microscopes and electron beam systems.

Only one paper was found that discusses construction vibration impacts on sensitive facilities requiring a VC A or tighter specification. Amick & Gendreau^8^ detail the vibratory effect and subsequent constraints on construction for a new building located next to an existing semiconductor manufacturing plant with a vibration category E specification, which specifies vibrations must be kept below an RMS particle velocity of 3 μm/s. An example for the specific site they were on in Silicon Valley, USA, they conducted experiments that found the setback distance at which a 25‐Hz compactor could be used was 85 m in order to keep the vibrations below category D (6 μm/s RMS particle velocity).

The concerns that arose directly from the vibrations were the effect on patient treatment specifically couch and linear accelerator (linac) motion during treatment and the effect on the setup laser accuracy, effect on staff and patients especially in terms of patient experience, trust and comfort, and the effect on the linac treatment units.

## 2 MATERIALS AND METHODS

The initial response to the vibrations was twofold: communication with the project management team to determine what was occurring and how long the vibrations would continue; and communication with the linac vendor, Varian Medial Systems (Palo Alto, CA, USA), to determine the implications for the treatment equipment.

At the start of the discussions with the project management team, they indicated that the piling was scheduled to take a minimum of 3 months. In response, a number of local measures were implemented immediately to mitigate most of the concerns stated above. The first response was to implement a weekly check of linac isocenter and laser alignment using a simplified Winston–Lutz test: using only images acquired at the cardinal gantry angles and an object located at the intersection of the setup lasers on Clinac units and for the Truebeam units performing the isocal verification procedure. While the vibrations were concerning, they were occurring randomly and for periods affecting only a small number of patients, this led the medical physics department to advise the therapists to continue treating patients with more than five fractions. However, we also gave the staff a point of contact with whom they could communicate with if they felt uncomfortable treating due to the vibrations. Stereotactic radiosurgery patient treatments were of particular concern due to high required accuracy. We attempted to coordinate treatments with the construction site to avoid vibrations during treatments and instituted a rapid response line. When vibrations were felt before or started during an SRS patient, the treatment was paused, and the construction site was contacted to halt activity until treatment was completed. These actions, along with open and continued communication, in turn allowed the treatment staff to communicate and reassure patients.

The center affected used only Varian linear accelerators (Varian Medical Systems, Palo Alto, CA). Discussions with Varian revealed that the linacs were unlikely to be affected functionally by the vibrations as they are earthquake rated. However, with vibrations like the ones we were reporting, there is cause for concern regarding the accuracy of patient treatment. The document Varian provided specified that for accurate patient treatment, the total patient‐field positioning error allowable is 0.1 mm zero‐to‐peak, incorporating both linac head and treatment couch motion. The general requirement is that the site to conform to VC‐D level (RMS velocity of 6 μm/s), or, if it does not, there is a method that can be used to determine the linac and couch motion that requires measurement of site‐specific displacement vs frequency. These data are acquired using an accelerometer that measures time resolved velocity (μm/s), which is converted by the datalogger into the frequency domain using a fast Fourier transform and finally to displacement (μm) through integration or acceleration (μm/s^2^) through differentiation.

The alternate method of determining the vibration suitability of the site takes the displacement vs frequency data described above and applies a resonant amplification factor to the amplitude as a function of frequency. The resonant amplification factor is derived from the resonance of the system component; treatment head at 0°, 90°, or the treatment couch. The consequence of this is that a small vibration at one of the resonant frequencies can cause significantly greater vibrations in the Linac system. The resonant amplification factors were part of the vendor supplied, proprietary documentation we requested to assess the impact on our units, and depend on model, manufacturer and gantry angle. Both the gantry and the couch had independent vibration responses, and both were considered in this work. The documentation demonstrated that the C‐Series and Truebeam systems responded differently both in terms of resonant frequencies and amplification ratio. For both systems, the couch was the main source of uncertainty due to resonance. There exist resonant peaks with amplification factors greater than 10 for both systems at frequencies from 2 Hz up to 35 Hz, with some maximums reaching factors of over 100 for each system.

We did not measure or investigate the vibratory characteristics of the source/s of the vibrations, but more relevantly we measured the propagated amplitudes and frequencies in the treatment vault. These were found to overlap with some, but not the worst, resonant frequencies of the linac gantry/couch systems. Centers dealing with vibration issues are advised to work with their specific vendor to obtain specifications for their current equipment.

**Figure 1:**
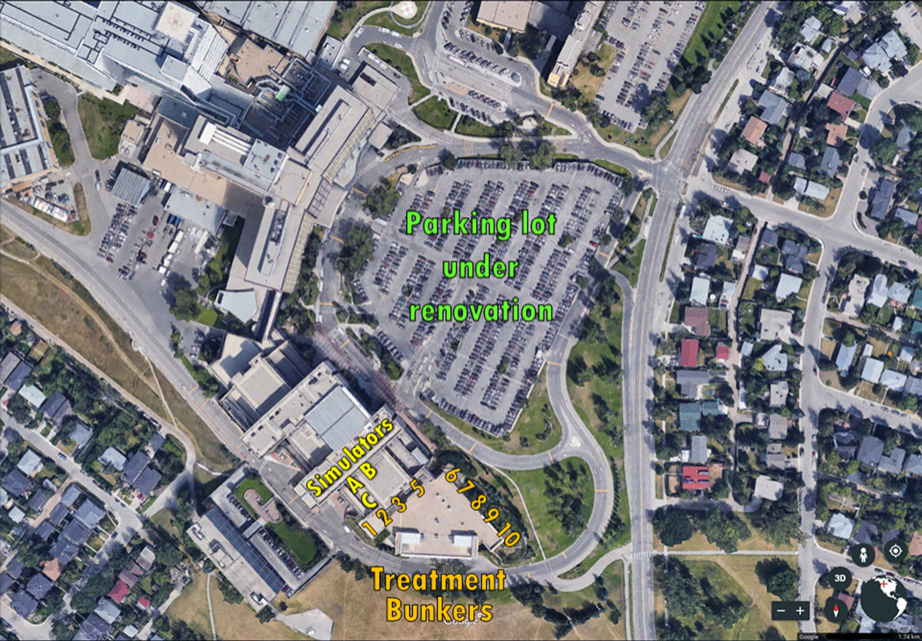
Location of construction site relative to TBCC. Treatment vaults are labeled numerically. The stereotactic radiosurgery (SRS) vault is #3. The location of the CT and conventional simulators is also indicated.

As the construction moved to casings closer to the cancer center, the vibrations became more strongly felt. In response, the department escalated to halting all patient treatments whenever significant vibrations were felt and site‐specific testing was performed. External consultants were engaged to measure the magnitude of the vibrations caused by the piling activities. The consultants provided a single‐axis accelerometer connected to a datalogger, which was capable of either passively logging average and maximum displacement every 30 s or performing a FFT on 8 s of acquired data (which could not be performed retrospectively). It was noted that certain areas of the rooms, not necessarily closest to the vibration source, experienced higher magnitude of vibrations. These areas of interest were detectable by the simple process of walking around the room while the vibrations were ongoing. The accelerometer was installed sequentially in two locations: on the floor in the control room (InCloset) and on the floor behind the linac stand in the treatment room (InRoom), both in Vault 6, which was the vault closest to the construction at that time (Fig. 1) and where the vibrations were most strongly felt. In our short time with the equipment, we were not able to determine if the areas of apparent maximum vibration moved when the location of the construction work outside moved, so a worst‐case scenario, that the maximum vibration in the vault would affect the linacs, rather than only measuring by the couch and machine base, was used.

The vibratory compactors also produced vibrations in the department that were detectable by staff and patients, but were far more transient in nature. By the time this phase of work was begun, the department no longer had access to the accelerometer to determine the amplitude as a function of frequency, so a simple test to determine acceptable vibration levels was required. The “Jurassic” test devised was to place a cup of water on the treatment couch and observe the surface of the water for ripples with the premise that problematic vibrations would cause the water surface to move.

## 3 RESULTS

**Figure 2:**
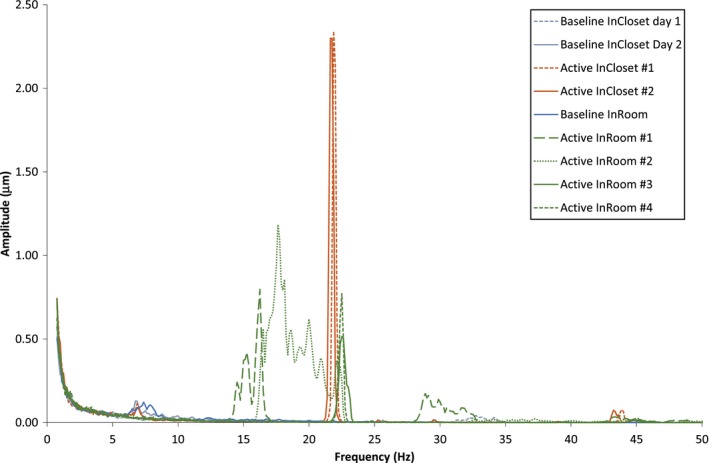
The average zero‐peak displacement over 20 individual 8‐s samples as a function of frequency, measured as background (no construction activity) and under active vibrations conditions in two locations chosen based on ease of access during treatment hours and length of time with the equipment. InCloset was on the floor in the control room and InRoom was on the floor behind the linac stand. Measurements were acquired over 2 days with the same equipment running (InCloset background on day 1, InCloset and InRoom background and under active conditions on day 2) and repeated approximately every 5 min for the duration of the vibration.

Using the accelerometer provided by the external consultants and communicating with the construction site, vibrational amplitude vs frequency data were acquired in the control and treatment rooms under background conditions and during active vibratory piling conditions. Each of the data tables provided by the consultant contained the average and maximum zero to peak displacement for frequencies between 1 and 50 Hz taken from 20 individual 8‐s samples. Figure 2 shows the frequency vs vibrational amplitude recorded by the accelerometer for baseline and under active vibratory piling conditions. Under active vibratory piling, resonant peaks were observed primarily between 20 and 25 Hz with average displacements over 20 samples up to 2.35 μm. The measurements in Fig. 2 were acquired during the operation of the same piece of equipment while it was in the vibration drilling phase. This phase lasted between 15 and 45 min per casing. The InCloset measurements were acquired under steady‐state conditions, and the InRoom measurements were acquired under both initial ramp up and steady‐state vibratory conditions.

The Winston‐Lutz isocenter vs setup laser and isocal verification tests were performed weekly on each treatment unit from mid‐November till late December when the method of piling was changed. No measurable change in linac position relative to either the couch or the setup lasers was detected using these tests. During periods of vibrations, the laser lines for patient setup could vibrate significantly, however. Postmounted lasers were more affected than wall mounted lasers. Our department stabilized the postmounted lasers in the affected vaults with attachments to the wall to reduce the movement of these during patient setup.

Ultimately, the limitations the department set on acceptable vibratory activity corresponded closely with what the majority of patients and staff could easily feel. Therefore, the consultant's advice of “if you can feel the vibrations, it is outside of specification” was used, for low‐frequency vibrations the threshold for feeling is in the region of 1–10 μm. This somewhat qualitative response accommodated the urgency of providing guidance to the construction site so work could continue.

**Figure 3:**
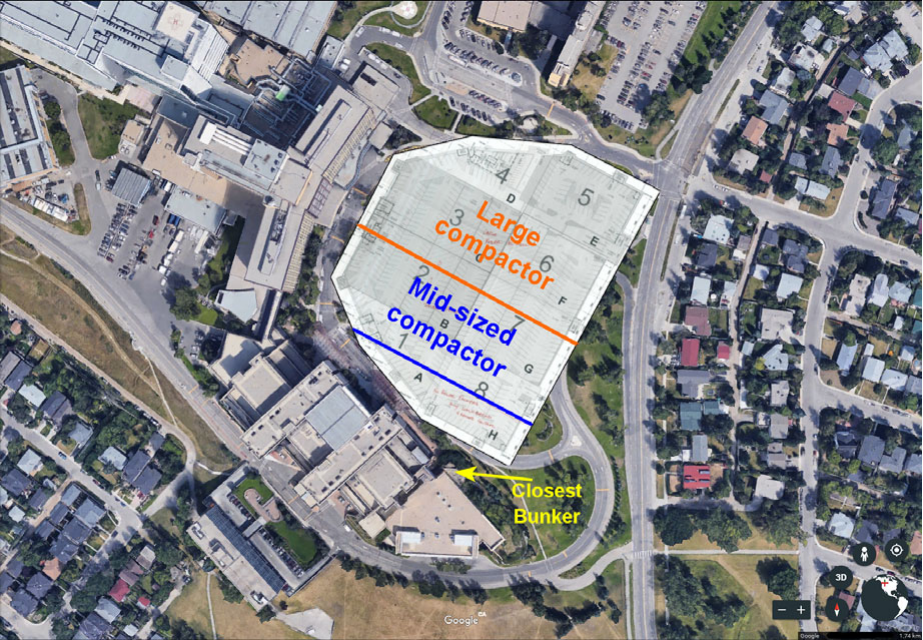
Satellite image of hospital site with parkade plans overlaid.

When vibratory compactors were identified as an issue in early 2017, this rule was used to determine acceptable operating setbacks. These tests were conducted one evening after treatments had finished. A cup of water was placed on the treatment couch of each affected treatment room. Each of the compactors was operated independently on the construction site and gradually driven further from the department until the vibrations no longer caused observable ripples in the water. The setbacks for vibratory compactors were approximately 30 m for the 500 lb compactor, 50 m for the midsized compactor, and 100 m for the 8000 lb compactor. A schematic of this is shown in Fig. 3.

## 4 DISCUSSION AND CONCLUSION

The analysis by the medical physics department and consultant, following review of the vendor vibration specification document, was, in simplest form, if you can feel it, do not treat until site‐specific testing has been conducted. This result depends on the transmitted frequency from construction and its relation to the resonant frequencies for the linacs and couches, which were unfortunately quite close at this site. Using the FFT data (Fig. 2) and the resonance amplitude method from the vendor specification document, the maximum total vibration under active vibratory piling of the treatment system was 268 μm for the Varian Clinac (minimum 38 μm of the measured points) and 143 μm for the Varian Truebeam (minimum 20* *μm). As the range of total vibration values above and in Fig. 2 demonstrate, there was large variation. The variation was caused by changes in the stage (ramp up or steady state) of the piling activity, the treatment head position (head up/down verses head 90°/270°) and the type of machine as well as the frequency of the peak amplitudes. A change of <1 Hz in the frequency of the peak amplitude could substantially reduce the resonant amplification factor. Vibration propagation through the ground, walls, floor, and equipment is nontrivial. The location of the maximum vibration within the room appeared to move around the building as work was conducted at different locations outside, and measurements at the different locations in the building had vastly different readings. Given the limited access to the accelerometer, our goal was to determine if a reasonable approximation of the maximum motion exceeded the manufacturer's specifications, rather than to fully characterize vibratory motion within the treatment vault and console.

As the maximum resonant vibration was greater than the vendor recommended limit of 100 μm, these results led to a revised recommendation from the medical physics department to the project management team that the vibration hammer was incompatible with radiotherapy treatment. Other options which were considered to facilitate the continued use of the vibration hammer during treatment were the addition of a “vibration” margin to treatment, coordination of piling such that treatment would pause while the vibratory hammer was in use and restriction of piling hours to outside of treatment hours. It proved very challenging to coordinate events on the construction site with patient treatments. The vibration hammer work to insert the casing could vary in length by more than a factor of 2, depending on the depth of the individual casing, the soil conditions, and so on. In addition, once the concrete was poured, the casing must be removed in a brief window after a set curing time. In December in Alberta, the weather is also a major complicating and constraining factor on major construction projects. Similarly, the exact time of patient treatments, particularly same day framed SRS patients, depended on a complex series of preparatory steps, so treatment delivery times could also only be predicted to a window. Non‐SRS patient challenges included chemo coordination and bi‐daily treatment timing. The potential for interruptions and delays on both sides meant that coordination simply was not feasible. Limiting use of the vibratory hammer outside of treatment hours only was immediately eliminated by the project management team because it was simply unworkable if the project was going to be delivered even close to schedule.

In the case of an extra margin, the issue was how big to make it and to which patients since the timing and length of the vibrations was random and uncoordinated. The extended time of construction (3 months) meant that all patients in the department could be affected, resulting in reduced geometric accuracy, but the number of affected fractions for each patient was impossible to predict. Patients reported feeling insecure lying on a vibrating couch, and their confidence in the treatment process was also considered. Restricting gantry angles to avoid the major resonance in the gantry would be restrictive and ineffective, since the couch contributes a significant component of the vibration.

On reporting this result to the project management team and hospital management, it was decided that the method of piling had to be changed, despite the increased cost to the project. The chosen replacement method was to use oscillated segmental casings which removed the need to use the vibratory hammer.

Regarding the vibratory compactors, the setbacks determined qualitatively were similar to those calculated by Amick & Gendreau.^8^ They determined, based on the site‐specific measurements acquired, for VC‐D the setback required was 65–165 m, depending on the type of compactor and vibrator frequency.

This issue posed significant challenges for the clinical and project teams. Vibration propagation through the soil, into buildings and its effects within the building is a complex problem, and accurate predictions are not always possible. Based on our experience, we recommend vigilance during construction as well as initiating during design phases of a project identifying this potential as a significant risk, developing and maintaining open lines of communication with the project team. A joint, collaborative approach seeking to maintain clinical treatments and project goals and timelines built goodwill for both sides and allowed the groups to navigate the difficult decisions together for the benefit of our patients.

## CONFLICT OF INTEREST

The authors declare no conflicts of interest.
